# Author Correction: Moist and warm conditions in Eurasia during the last glacial of the Middle Pleistocene Transition

**DOI:** 10.1038/s41467-025-57243-5

**Published:** 2025-02-24

**Authors:** María Fernanda Sánchez Goñi, Thomas Extier, Josué M. Polanco-Martínez, Coralie Zorzi, Teresa Rodrigues, André Bahr

**Affiliations:** 1 Ecole Pratique des Hautes Etudes (EPHE, PSL University), Paris, France; 2 Univ. Bordeaux, CNRS, Bordeaux INP, EPOC, UMR 5805, 33600 Pessac, France; 3 Unit of Excellence GECOS, IME, University of Salamanca, 37007 Salamanca, Spain; 4 Basque Centre for Climate Change (BC3), 48940 Leioa, Spain; 5 Divisão de Geologia e Georecursos Marinhos, Instituto Português do Mar e da Atmosfera, Rua Alfredo Magalhães Ramalho, 6, 1495-006 Lisboa, Portugal; 6 Institute of Earth Sciences, Heidelberg University, Im Neuenheimer Feld, 234, 69120 Heidelberg, Germany

Correction to: *Nature Communications* 10.1038/s41467-023-38337-4, published online 10 May 2023

In the version of the article initially published, there was a typo in the last sentence of the Results where, in the sentence now reading “The long-term increasing T_therm_ SST gradient trend in the North Atlantic…,” increasing originally read “decreasing.” The text is amended in the HTML and PDF versions of the article.

