# Supplementary material for: Moist and warm conditions in Eurasia during the last glacial of the Middle Pleistocene Transition
Source: Nat Commun. 2023 May 10;14:2700. doi: 10.1038/s41467-023-38337-4 (PMC10172347; doi:10.1038/s41467-023-38337-4)
Supplement: Supplementary file 1 — Supplementary information [file 41467_2023_38337_MOESM1_ESM.pdf]

Supplementary Information

Moist and warm conditions in Eurasia during the last glacial of the Middle Pleistocene Transition

María F. Sánchez Goñi[1,2] *, Thomas Extier[2], Josué M. Polanco-Martínez[3,4], Coralie Zorzi[2], Teresa Rodrigues[5], André Bahr[6]

[1] Ecole Pratique des Hautes Etudes (EPHE, PSL University), Paris, France

[2] Univ. Bordeaux, CNRS, Bordeaux INP, EPOC, UMR 5805, F-33600 Pessac, France

[3] Unit of Excellence GECOS, IME, University of Salamanca, E 37007 Salamanca, Spain

[4] Basque Centre for Climate Change (BC3), 48940 Leioa, Spain

[5] Divisão de Geologia e Georecursos Marinhos, Instituto Português do Mar e da Atmosfera, Rua Alfredo Magalhães Ramalho, 6, 1495-006 Lisboa, Portugal

[6] Institute of Earth Sciences, Heidelberg University, Im Neuenheimer Feld, 234, 69120 Heidelberg, Germany

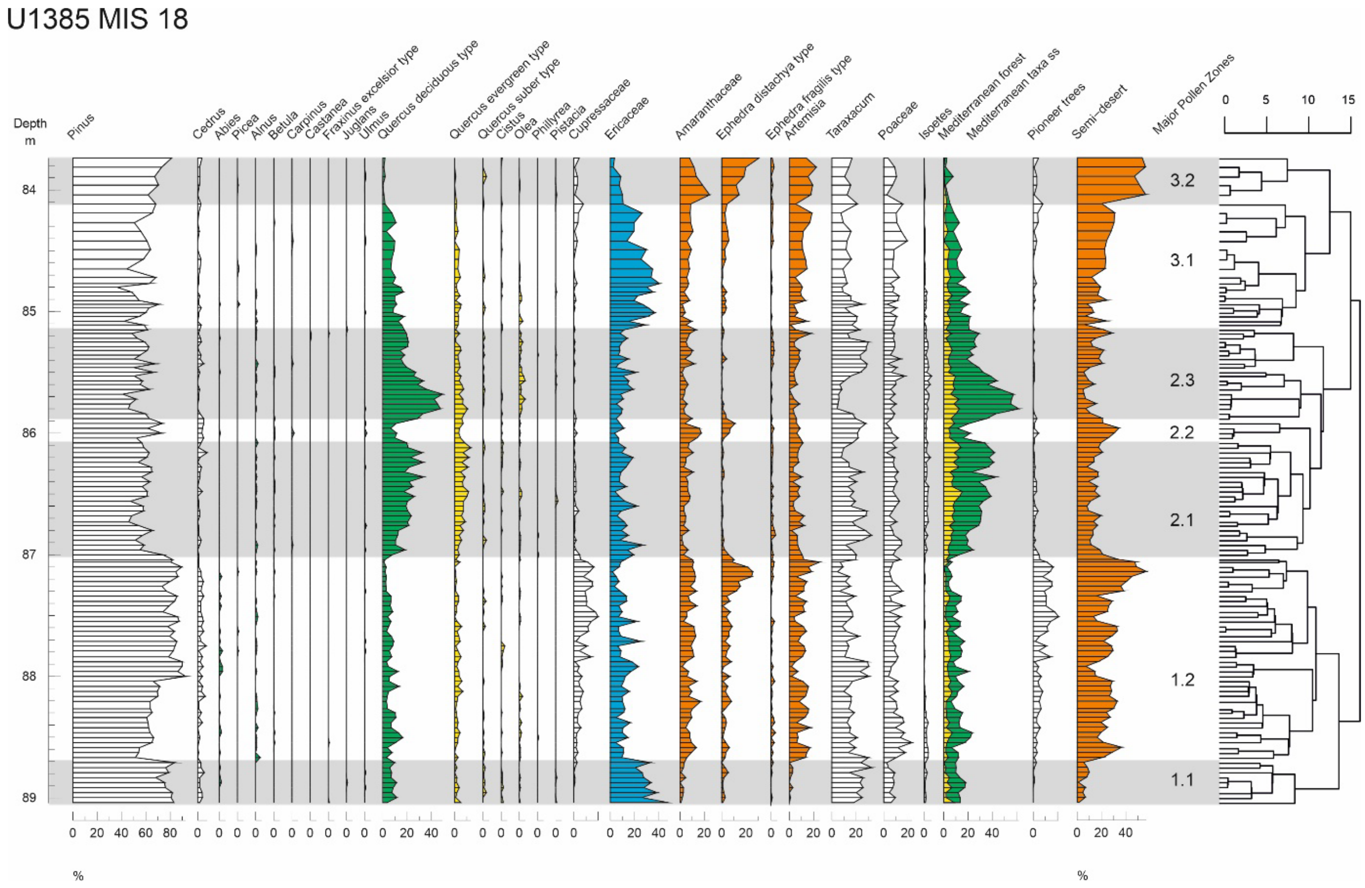

Figure S1 – Pollen diagram of MIS 18 from IODP Site U1385 with selected taxa only. The most representative taxa for the Mediterranean forest (green) are *Alnus, Betula, Ulmus* and deciduous *Quercus*, including the Mediterranean taxa (yellow, sclerophyllous) that are evergreen *Quercus, Cistus, Olea, Phillyrea* and *Pistacia.* Semi-desert group includes Amaranthaceae, *Artemisia*, *Ephedra dystachia*-type and *E. fragilis*-type (orange). Ericaceae family includes *Calluna* and represents the heathlands (blue). Cluster analysis is represented on the right side showing major palynological zones.

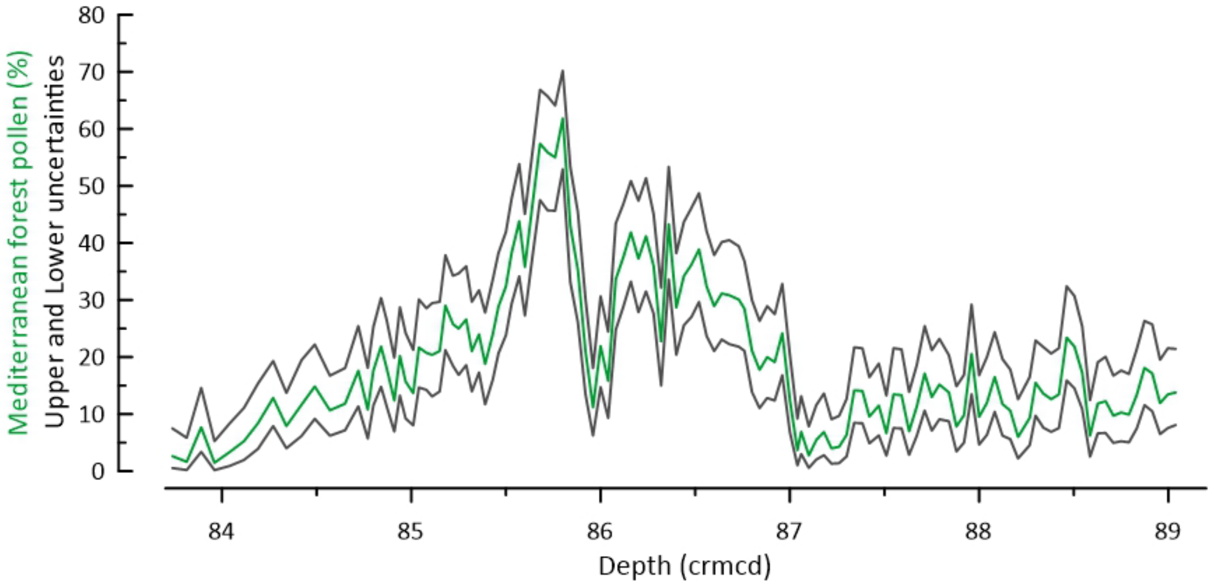

Figure S2 – Uncertainties (grey) in the Mediterranean pollen percentages (green) at 95% confidence interval. crmcd – corrected revised composite depth scale.

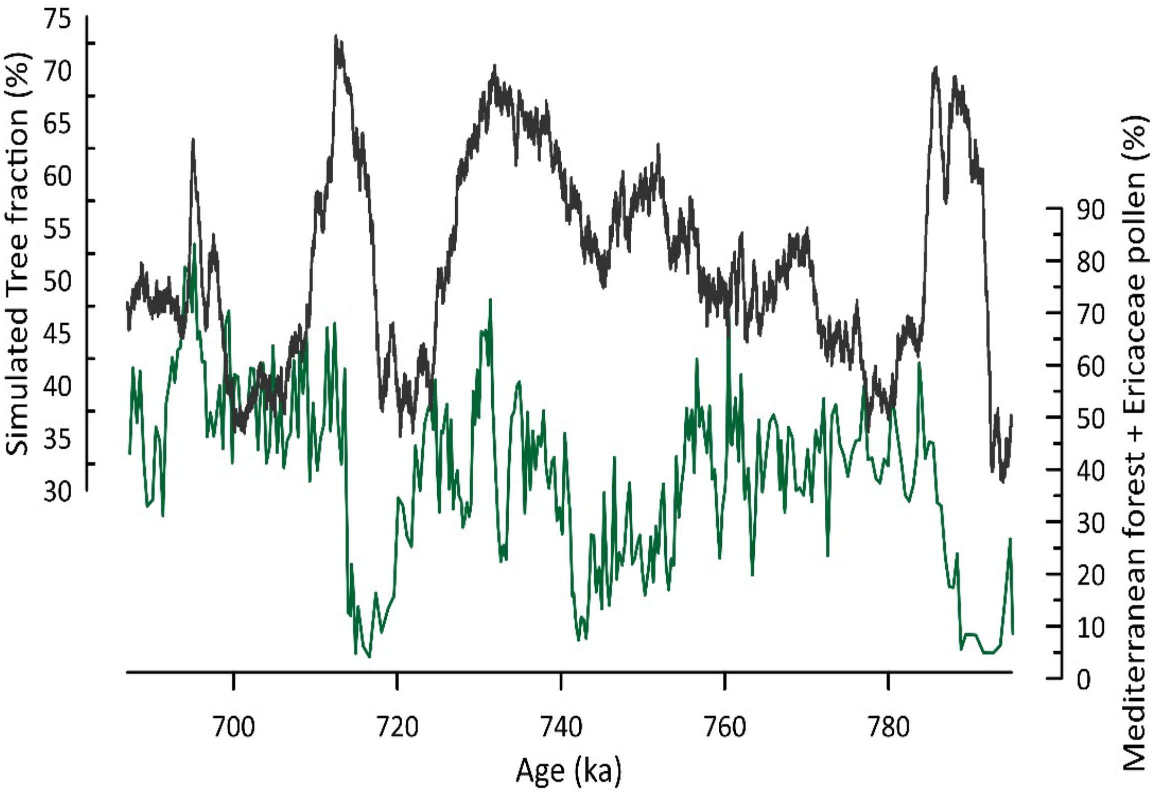

Figure S3 – Comparison between the simulated tree fraction (black), including trees and shrub species, and the Mediterranean forest plus Ericaceae pollen percentages (green) across MIS 19 to 17.

a

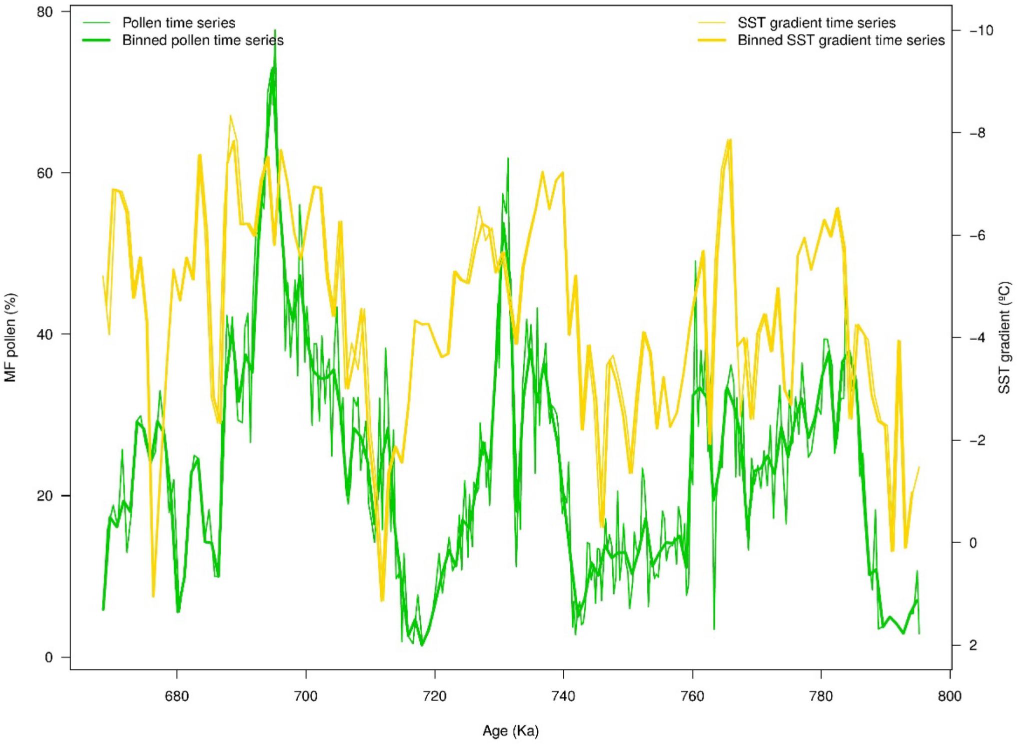
Pollen time series
Binned pollen time series
SST gradient time series
Binned SST gradient time series
MF pollen (%)
SST gradient (ºC)
Age (Ka)
80
60
40
20
0
−10
−8
−6
−4
−2
0
2
680
700
720
740
760
780
800

b

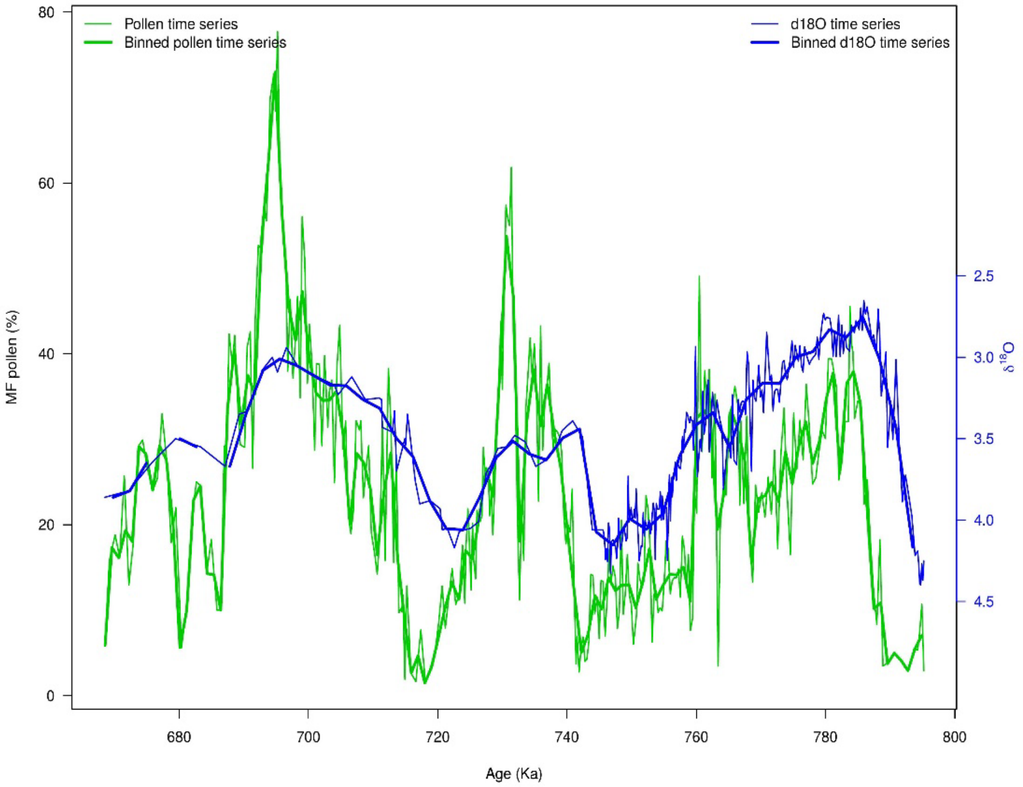
Pollen time series
Binned pollen time series
d18O time series
Binned d18O time series
MF pollen (%)
δ18O
Age (Ka)
80
60
40
20
0
2.5
3.0
3.5
4.0
4.5
680
700
720
740
760
780
800

c

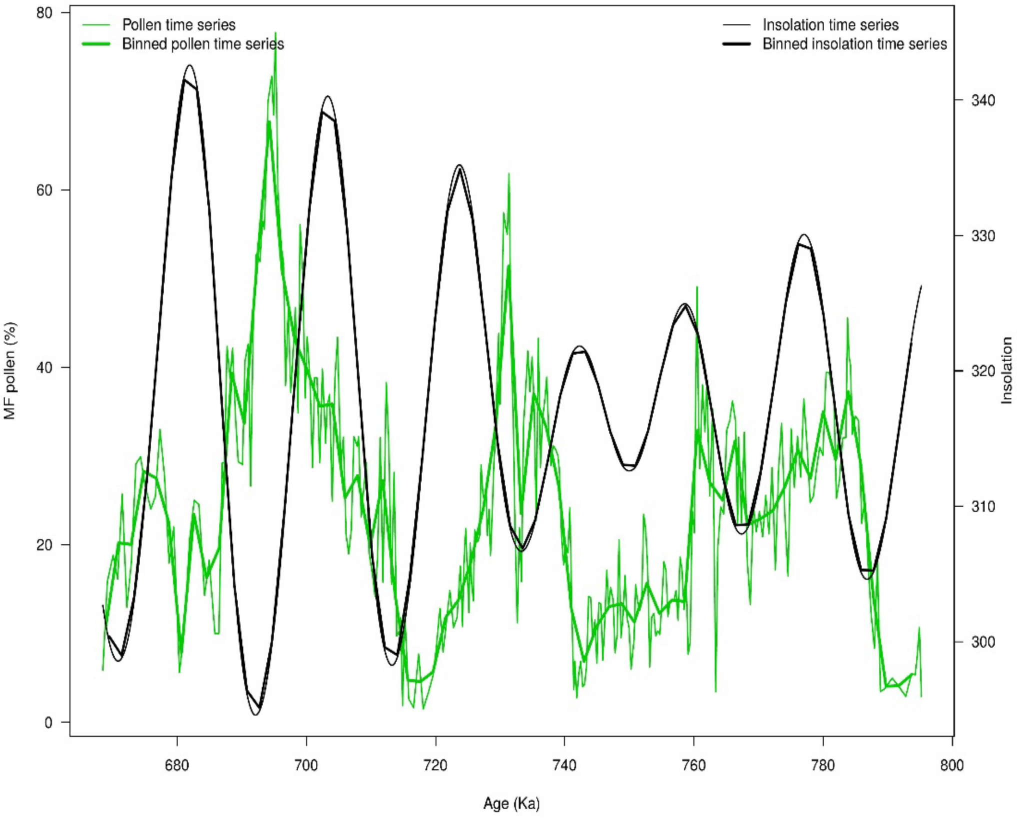

Figure S4. Original (unevenly spaced) and binned paleoclimate time series under study: **a** T_{therm} SST (sea surface temperature) gradient vs Mediterranean forest (MF), **b** benthic foraminifera $\delta^{18}O$ vs Mediterranean forest, and **c** winter latitudinal insolation gradients Mediterranean forest. The persistence values (in Ka) for the SST gradient and MF for the original and binned series are 2.379 and 2.526 and 3.038 and 7.436; the persistence values for the benthic foraminifera $\delta^{18}O$ and MF for the original and binned series are 12.942 and 3.038 and 22.087 and 12.633; and for the insolation gradient are 56.859 and 3.038 and 19.630 and 8.326. The number of data points and the bin width (in Ka) for the T_{therm} SST gradient and MF are 121 and 1.05; for the benthic foraminifera $\delta^{18}O$ and MF are 47 and 2.588; and for the insolation and MF are 65 and 1.947.

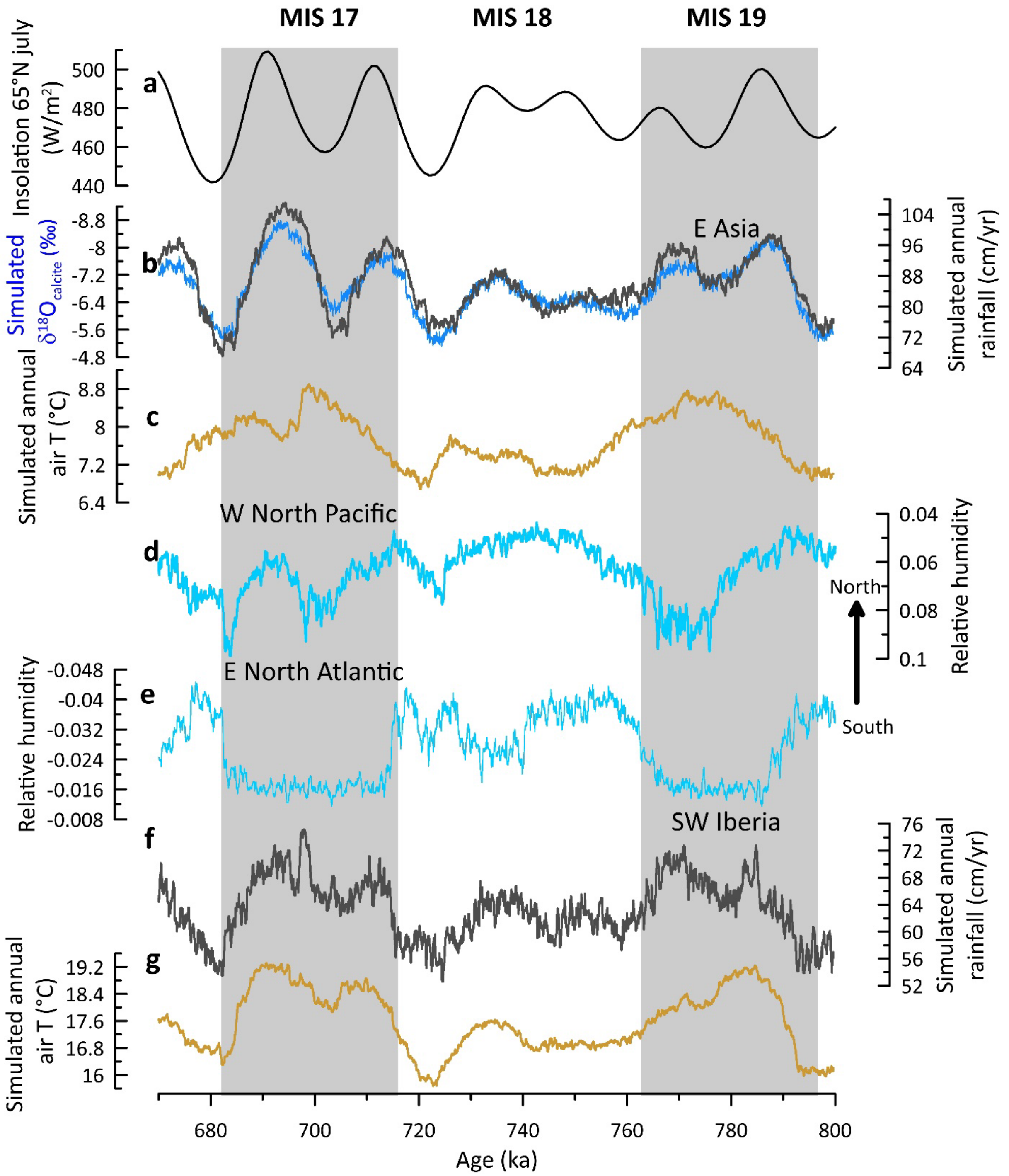

Figure S5 – iLOVECLIM simulation results across MIS 19 to MIS 17 interval (this study). **a** July insolation at 65°N [1], **b** annual rainfall in East China (black) and $\delta^{18}O_{calcite}$ (blue), **c** annual air temperature at 2 m in East China, **d** relative humidity between ODP Sites 882 and 1143 indicating the location of the moisture source in the West North Pacific, **e** relative humidity between IODP Sites U1313 and U1385 indicating the location of the moisture source in the eastern North Atlantic, **f** annual rainfall in South-West Iberia, **g** annual air temperature at 2 m in South-West Iberia.

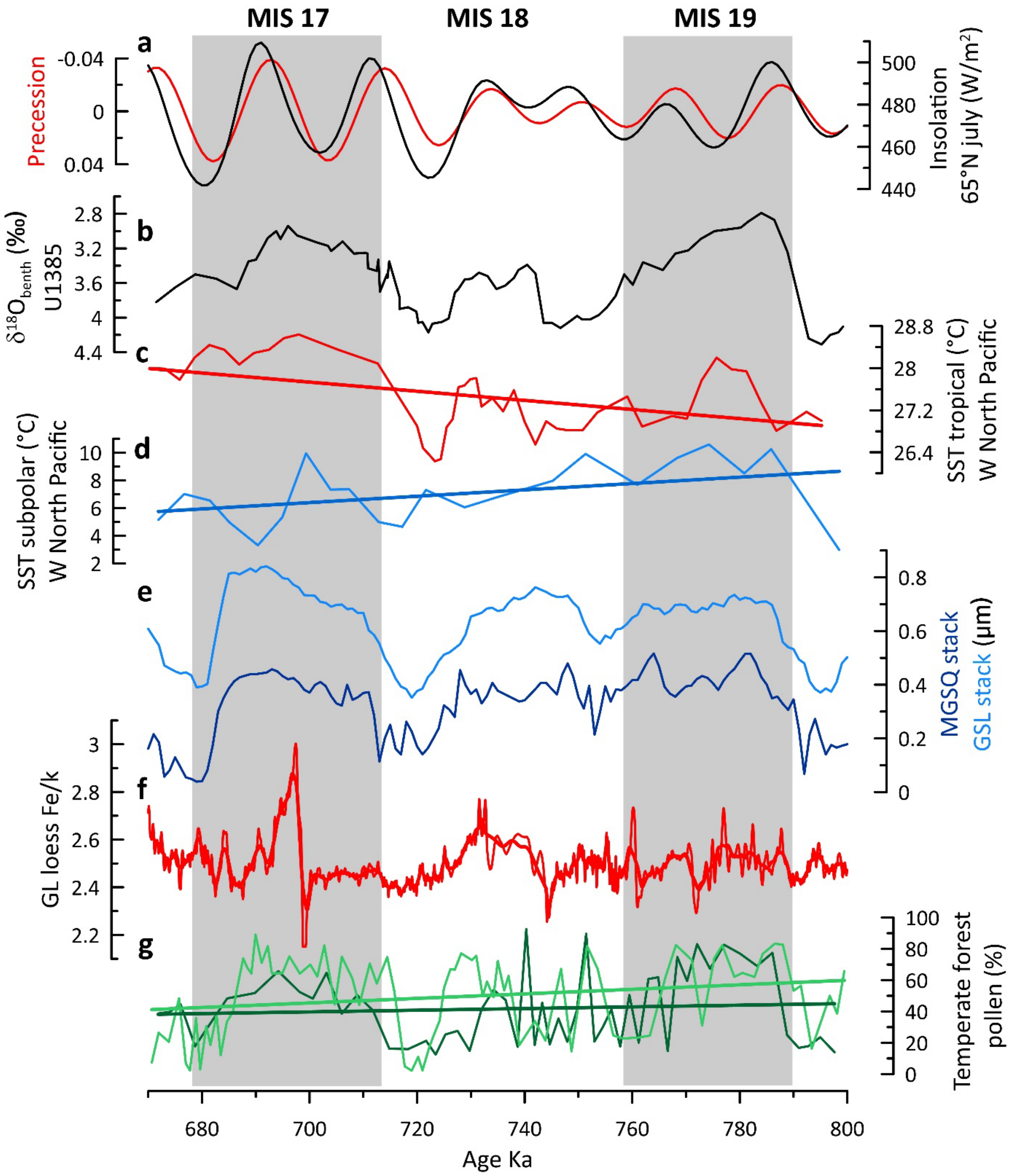

Figure S6 – Paleoclimatic records from Eastern North Atlantic margin, Europe, the Chinese Loess Plateau and West North Pacific. **a** Insolation at 65°N in July (black), and precession (red) [1], **b** $\delta^{18}O$ of benthic foraminifera from IODP Site U1385 [2], **c** SST record from ODP Site 1143 located in the tropical West North Pacific Ocean [3], **d** SST record from ODP Site 882 located in the subpolar West North Pacific Ocean [4], **e** Mean Grain Size Quartz (MGSQ) stack from Lingtai, Zhaojiachuan, Pingliang, Baoji, Luochuan, Puxian, Jingbian and Jiaxian sequences (dark blue) [5] and Grain Size Loess (GSL) stack from Pingliang, Jingshuan, Baoji, Lingtai, Puxian sequences, both stacks indicate changes in East Asia winter monsoon (EAWM) [6], **f** Fe/K record from the Chinese Loess Plateau Gulang sequence indicating

changes in East Asia summer monsoon (EASM) [7], **g** Temperate forest pollen percentage records from Tenaghi Philippon (North-East Greece, light green) [8] and Lake Ohrid (North Macedonia/Albania, dark green) sedimentary sequences [9]. In Lake Ohrid, “Temperate forest” includes the total trees excluding Pinus, i.e. montane trees (*Abies, Betula, Cedrus, Fagus, Ilex, Picea, Taxus, Tsuga*); mesophyllous trees (*Acer, Buxus, Carpinus betulus, Carya, Castanea, Celtis, Corylus, Fraxinus excelsior/oxycarpa, Hedera, Ostrya/Carpinus orientalis, Pterocarya, Q. cerris–type, Q. robur–type, Tilia, Ulmus, Zelkova*); Sclerophyllous trees (*Arbutus, Cistus, Fraxinus ornus, Olea, Phillyrea, Pistacia, Quercus ilex–type, Rhamnus*), and wetland trees (*Alnus, Liquidambar, Platanus, Populus, Salix, Tamarix, Taxodium*-type). In Tenaghi Philippon, « Temperate forest » includes the total trees pollen percentages including *Pinus* [8]. The shape of the total trees pollen percentage curve excluding *Pinus* is not substantially different from that shown in [9]. Grey bands indicate the Marine Isotope Stages (MIS) 19 to 17. Panels c, d, g: straight lines indicate ordinary least squares fits. Panel f: thick line indicate weighted average fit with a 23-sample window

Supplementary References

1 Laskar, J. *et al.* A long-term numerical solution for the insolation quantities of the Earth. *A&A* **428**, 261-285 (2004).

2 Hodell, D. A. *et al.* A reference time scale for site U1385 (Shackleton Site) on the Iberian Margin. *Global and Planetary Change* **133**, 49-64 (2015).

3 Martínez-Garcia, A., Rosell-Melé, A., McClymont, E. L., Gersonde, R. & Haug, G. H. Subpolar Link to the Emergence of the Modern Equatorial Pacific Cold Tongue. *Science* **328**, 1550-1553, doi:doi:10.1126/science.1184480 (2010).

4 Li, L. *et al.* A 4-Ma record of thermal evolution in the tropical western Pacific and its implications on climate change. *Earth and Planetary Science Letters* **309**, 10-20, doi:https://doi.org/10.1016/j.epsl.2011.04.016 (2011).

5 Sun, Y., Clemens, S. C., An, Z. & Yu, Z. Astronomical timescale and palaeoclimatic implication of stacked 3.6-Myr monsoon records from the Chinese Loess Plateau. *Quaternary Science Reviews* **25**, 33-48, doi:https://doi.org/10.1016/j.quascirev.2005.07.005 (2006).

6 Ding, Z. L. *et al.* Stacked 2.6-Ma grain size record from the Chinese loess based on five sections and correlation with the deep-sea δ18O record. *Paleoceanography* **17**, 5-1-5-21, doi:https://doi.org/10.1029/2001PA000725 (2002).

7 Sun, Y. *et al.* Persistent orbital influence on millennial climate variability through the Pleistocene. *Nature Geoscience* **14**, 812-818, doi:10.1038/s41561-021-00794-1 (2021).

8 Tzedakis, P. C., Hooghiemstra, H. & Pälike, H. The last 1.35 million years at Tenaghi Philippon: revised chronostratigraphy and long-term vegetation trends. *Quaternary Science Reviews* **25**, 3416-3430, doi:http://dx.doi.org/10.1016/j.quascirev.2006.09.002 (2006).

9 Donders, T. *et al.* 1.36 million years of Mediterranean forest refugium dynamics in response to glacial–interglacial cycle strength. *Proceedings of the National Academy of Sciences* **118**, e2026111118 (2021).

```r
#######################################################################
#:: bin_cor function - R package BINCOR                               #
#:: Programmed by Josué M. Polanco-Martinez a.k.a jomopo              #
#:: Email: josue.m.polanco@gmail.com                                  #
#######################################################################
#   Copyright (C) 2017, 2023 by Josué M. Polanco-Martínez             #
#   This file/code is part of the R package BINCOR                    #
#######################################################################
#
#   BINCOR is free software: you can redistribute it and/or modify it
#   it under the terms of the GNU General Public License as published
#   by the Free Software Foundation, either version 3 of the License,
#   or (at your option) any later version.
#
#   BINCOR is distributed in the hope that it will be useful,
#   but WITHOUT ANY WARRANTY; without even the implied warranty of
#   MERCHANTABILITY or FITNESS FOR A PARTICULAR PURPOSE.  See the
#   GNU General Public License for more details.
#
#   You should have received a copy of the GNU General Public License
#   along with BINCOR If not, see <http://www.gnu.org/licenses/>.
#
######################################################################

bin_cor <- function(ts1, ts2, FLAGTAU=3, rho=NULL, rho1=NULL, tau1=NULL,
                    rho2=NULL, tau2=NULL, ofilename) {

 #:: inputs:
 #:: ts1 and ts2 are the time series under analysis (the first column
 #:: are the times/ages (in ascending order) & the second column are the
 #:: elements of the variable under stiudy). "ofilename" is the output's
 #:: filename, which will contains the binned data.
 #:: FLAGTAU (the persistence method).
 #:: Options (by default it is 3):
 #:: 1 tau_x + tau_y     [Eq. 7.44, Mudelsee (2010, 2014)]
 #:: 2 max(tau_x, tau_y) [Eq. 7.45, Mudelsee (2010, 2014)]
 #:: 3 dist_x_y/ln(a_x_y_est) [Eq. 7.48, Mudelsee (2010, 2014)]
 #:: tau_x, tau_y are the persistence (memory) time for ts1 and ts2,
 #:: respectively.

 #:: Checking the input data
 if(dim(ts1)[2] !=2 | dim(ts2)[2] != 2)
  stop ("There is a problem with the dimension in your input data.
   The input data should be a couple of vectors of dimension N x 2
   (rows x columns). Thank you for using the BINCOR package. \n")

 if(length(which(diff(ts1[,1]) <= 0)) | length(which(diff(ts2[,1]) <= 0)))
  stop ("There are some times/ages that are not strictly monotonic ascending.
  Please, check your data. Thank you for using the BINCOR package. \n")

 if(all.equal(ts1[,1], ts2[,1]) == "TRUE")
  cat("The time series have the same timescales, it's not necessary to
   perform the binned procedure, but it will be computed. \n")

 #:: Getting the names of the variables
 names.ts1 <- names(ts1)
 names.ts2 <- names(ts2)
 if(is.null(colnames(ts1))) colnames(ts1) <- c("Time-ts1", "Variable-ts1")
 if(is.null(colnames(ts2))) colnames(ts2) <- c("Time-ts2", "Variable-ts2")

 #:: Getting Nx & Ny (number of elements for each time series)
 Nx     <- length(ts1[,1])
 Ny     <- length(ts2[,1])

 #:: Computing Eqs. 7.46 (Mudelsee 2010, 2014)   # or smarter in R ;-)
 dist_X  <- (ts1[Nx,1] - ts1[1,1])/ (Nx - 1)     # mean(diff(ts"x"[,1]))
 dist_Y  <- (ts2[Ny,1] - ts2[1,1])/ (Ny - 1)
 Tmax_m  <- max(ts1[Nx,1], ts2[Ny,1])
 Tmin_m  <- min(ts1[1,1],  ts2[1,1])
 dist_XY <- (Tmax_m - Tmin_m) / (Nx + Ny - 1)

 #:: Getting Tau (persistence time or memory for each t.s.)
 #:: There are several ways to get 'tau', but we use Mudelsee (2002).
```

```
 #:: This tau estimation can be obtained in R from the REDFIT (Schulz
 #:: & Mudelsee 2002) function included in the 'dplR' R package
 #:: (Bunn et al 2015. https://cran.r-project.org/package=dplR).
 #:: We compute the raw spectrum (dof=2) for each time series (n50=1 & iwin=0)
 #:: in order to get tau (subroutine redfitTauest), we have modified slightly
 #:: the version of the redfit subroutine redfitTauest. This piece of
 #:: code ("tauest_dplR.R") is provided in our BINCOR package.

########################################################################
########################################################################
if(0){
 #tau1 <- unlist(redfitTauest(ts1[,1], ts1[,2])) #If you use tauest_dplR.R
 #tau2 <- unlist(redfitTauest(ts2[,1], ts2[,2]))

 #a_X_est   <- tau1[2]
 #a_Y_est   <- tau2[2]
 #tau_X_est <- tau1[3]
 #tau_Y_est <- tau2[3]

 #If you use subroutine redfitTauest from 'dplR' R package
 redfit.ts1 <- redfit(ts1[,2], ts1[,1], ofac=1, n50=1, iwin=0, rhopre=rho)
 # for insolation
 #redfit.ts1 <- redfit(ts1[,2], ts1[,1], ofac=1, n50=1, iwin=0, rhopre=0.996472105)
 redfit.ts2 <- redfit(ts2[,2], ts2[,1], ofac=1, n50=1, iwin=0, rhopre=rho)
 #tau1       <- redfit.ts1$tau
 #tau2       <- redfit.ts2$tau

 a_X_est   <- redfit.ts1$rho
 a_Y_est   <- redfit.ts2$rho

 tau_X_est <- redfit.ts1$tau
 tau_Y_est <- redfit.ts2$tau
}

 a_X_est   <- rho1
 a_Y_est   <- rho2
 tau_X_est <- tau1
 tau_Y_est <- tau2

########################################################################
########################################################################

 #:: FLAGTAU (persistence for both time series) options!
 if (FLAGTAU == 1) {
  #:: Eq. 7.44 (Mudelsee 2010 & 2014).
  taub <- tau_X_est + tau_Y_est
  cat("Hi!, option 1: taub <- tau_X_est + tau_Y_est [Eq. 7.44 (Mudelsee 2010 & 2014)
] \n")
 }

 if (FLAGTAU == 2) {
  #:: Eq. 7.45 (Mudelsee 2010 & 2014)
  taub <- max(tau_X_est, tau_Y_est)
  cat("Hi!, option 2: taub <- max(tau_X_est, tau_Y_est) [Eq. 7.45 (Mudelsee 2010 & 2
014)] \n")
 }

 if (FLAGTAU == 3) {
  #:: Eq. 7.47 (Mudelsee 2010 & 2014)
  a_XY_est  <- sqrt(a_X_est*a_Y_est)
  #:: Eq. 7.48 (Mudelsee 2010 & 2014)
  taub      <- -dist_XY / log( a_XY_est )
  cat("Hi!, option 3: taub <- -dist_XY / log(a_XY_est) [Eq. 7.47 & 7.48 (Mudelsee 20
10 & 2014)] \n")
 }

 # Carlstein adaptation
 if (FLAGTAU == 4) {
  onethird=1/3
  twothird=2/3
  a_XY_est  <- sqrt(a_X_est*a_Y_est)
  dummy1    <- (sqrt(6))*a_XY_est
  dummy2    <- 1 - a_XY_est^2
```

```
 dummy3   <- (dummy1/dummy2)**twothird*Nx**onethird
 dummy3   <- max(1, dummy3)
 dummy3   <- max(Nx-1, dummy3)
 ###### Nx ??????
 taub     <- dummy3*(Tmax_m - Tmin_m)/( 2*Nx - 1)
}


#:: Inspired from the M. Mudelsee's code "mc-brxy.f90")
taub <- min(taub, (Tmax_m - Tmin_m)*0.5)
taub <- max(taub, (Tmax_m - Tmin_m)/(Nx - 1))

#:: Computing the number of "bins"
remi <- (Tmax_m - Tmin_m) / taub
Nb   <- round(remi)

cat("Testing the number of bins: taub=", taub," Nb=", Nb,"\n")

id1        <- rep(9999, Nb)
id2        <- rep(9999, Nb)
mean.ts1   <- rep(9999, Nb)
mean.ts2   <- rep(9999, Nb)
tau.t.mean <- rep(9999, Nb)
limI       <- Tmin_m

#:: Here, the binned time series are created!
for (N in 1:Nb) {
 limS  <- Tmin_m + N*taub
 if (N == 1) {
  id1t <- which(ts1[,1] >= limI & ts1[,1] <= limS)
  id2t <- which(ts2[,1] >= limI & ts2[,1] <= limS)
 }
 if (N > 1)  {
  id1t <- which(ts1[,1] >  limI & ts1[,1] <= limS)
  id2t <- which(ts2[,1] >  limI & ts2[,1] <= limS)
 }
 id1[N] <- length(id1t)
 id2[N] <- length(id2t)

 #:: Evaluating IF a "bin" contains BOTH more than zero
 #:: X (ts1) & Y (ts2) points (pp. 312, Mudelsee 2010)
 if (id1[N] & id2[N] > 0) {
  mean.ts1[N]   <- mean(ts1[id1t,2])
  mean.ts2[N]   <- mean(ts2[id2t,2])
  tau.t.mean[N] <- mean(c(limI, limS))  #or (limI + limS)/2
 }
 else {
 #:: This's a simple way to face this "problem", but you need to remove
 #:: the NA's to estimate the correlation btw "bin ts1" and "bin ts2".
  mean.ts1[N]   <- NA
  mean.ts2[N]   <- NA
  tau.t.mean[N] <- mean(c(limI, limS))  #(limI + limS)/2
 }
 limI   <- limS
}

Datin    <- cbind(tau.t.mean, mean.ts1, mean.ts2)
# mean.ts1 and mean.ts2 are the binned time series

#:: Computing some basic statistics
id.noNA     <- which(tau.t.mean != "NA")
avg.bin     <- round(mean(diff(tau.t.mean[id.noNA])), 2)
#avg.bin     <- mean(diff(na.omit(tau.t.mean)))
VAR.ts1     <- round(cbind(var(ts1[,2]), var(na.omit(mean.ts1))), 2)
VAR.ts2     <- round(cbind(var(ts2[,2]), var(na.omit(mean.ts2))), 2)
chg.VARts1  <- round(VAR.ts1[1] - VAR.ts1[2], 2)
chg.VARts2  <- round(VAR.ts2[1] - VAR.ts2[2], 2)
per_chg.VARts1 <- round((chg.VARts1 / VAR.ts1[1])*100, 2)
per_chg.VARts2 <- round((chg.VARts2 / VAR.ts2[1])*100, 2)


write.table(Datin, file=ofilename, col.names=F, row.names=F)
```

```
 names.ls <- c("Binned_time_series", "Auto._cor._coef._ts1", "Persistence_ts1",
               "Auto._cor._coef._ts2", "Persistence_ts2", "bin width", "Number_of_bi
ns",
               "Average spacing", "VAR. ts1", "VAR. bin ts1", "VAR. ts2", "VAR. bin
ts2",
               "VAR. ts1 - VAR bints1", "VAR. ts2 - VAR bints2", "% of VAR. lost ts1
",
               "% of VAR. lost ts2")

 LIST        <- list(Datin, a_X_est, tau_X_est, a_Y_est, tau_Y_est, taub, Nb,
                     avg.bin, VAR.ts1[1], VAR.ts1[2], VAR.ts2[1], VAR.ts2[2],
                     chg.VARts1, chg.VARts2, per_chg.VARts1, per_chg.VARts2)
 names(LIST) <- names.ls

 return(LIST)

}
```

```
#######################################################################
# This script is used to compute BINCOR correlation for this data set
# "Comparison_4variab_MIS-MIS18-MIS19.xlsx" (M.F. Sánchez Goñi), and
# to produce Figure S4 and used in "Moist and warm conditions in Eurasia
# during the last glacial of the Middle Pleistocene Transition", by
# MF Sánchez Goñi, T Extier, JM Polanco-Martínez, C Zorzi, T Rodrigues,
# A Bahr (2023), Nature Communications.
#######################################################################
# R Code written by Josue Polanco-Martinez (josue.m.polanco@gmail.com)
# Apr, 2023, Portugalete, Vizcaya, Spain
#######################################################################

 # To remove all object in the R session
 rm(list=ls())

 # Loading packages
 library("openxlsx")
 # BINCOR
 source("bin_cor_function.R")

 # Reading data
 datin <- list()
 for (i in 1:4) {
  datin[[i]] <- read.xlsx("Comparison_4variab_MIS17-MIS18-MIS19_JMPM.xlsx",
                sheet=i, skipEmptyRows=T, check.names=FALSE)
 }

 # Choosing MIS data from datin
 MIS_17_18_19 <- datin[[4]]
 MIS          <- MIS_17_18_19

 MF    <- as.ts(na.omit(cbind(MIS[,1], MIS[,2])))
 Inso  <- as.ts(na.omit(cbind(MIS[,3], MIS[,4])))
 Grad  <- as.ts(na.omit(cbind(MIS[,5], MIS[,6])))
 d18O  <- as.ts(na.omit(cbind(MIS[,7], MIS[,8])))

 #####################################################################
 # VERY IMPORTANT INFORMATION: tau1 and tau2 (persistence) were
 # computed via REDFIT (Schulz and Mudelsee 2002):
 # https://doi.org/10.1016/S0098-3004(01)00044-9
 # rho1 and rho2 (autocorrelation coefficient) is computed via acf
 # R function.
 #####################################################################

 bincor_Grad_MF <- bin_cor(Grad, MF, FLAGTAU=3, rho1=0.6569, tau1=2.3796,
  rho2=0.8737, tau2=3.03774, ofilename="binnedts_Grad_MF.dat")
 # Plot MF: raw + binned
 plot(MF[,1], MF[,2], t="l", xlab="Age (Ka)", ylab="MF pollen (%)", las=1)
 points(bincor_Grad_MF$Binned_time_series[,1],
  bincor_Grad_MF$Binned_time_series[,3], t="l", lwd=3)
 legend(bty="n", "topright", lty=rep(1,2), lwd=c(1.5,3),
  legend=c("Pollen time series", "Binned time series"))
 # Plot Gradient: raw + binned
 plot(c(Grad[,1]), c(Grad[,2]), xlab="Age (Ka)", ylab="SST Gradient (°C)",
  las=1, t="l", col=2)
 points(bincor_Grad_MF$Binned_time_series[,1],
  bincor_Grad_MF$Binned_time_series[,2], t="l", col=2, lwd=2.5)
 legend(bty="n", "topright", lty=rep(1,2), lwd=c(1.5,3), col=rep(2,2),
  legend=c("SST Gradient time series", "Binned SST Gradient time series"))
 # Overplot
pdf("plot_MF_Grad_original_binned_ts.pdf", width=12, height=9)
 par(oma=c(0, 0, 0, 1), mar=c(4.5, 4.5, 2, 3.5) + 0.1)
 plot(MF[,1], MF[,2], t="l", xlab="Age (Ka)", ylab="MF pollen (%)",
  las=1, lwd=1.5, col=3)
 points(bincor_Grad_MF$Binned_time_series[,1],
  bincor_Grad_MF$Binned_time_series[,3], t="l", lwd=3, col=3)
 par(new=T)
 plot(c(Grad[,1]), c(Grad[,2]), xlab="Age (Ka)", ylab="", las=1, t="l",
  col="gold", lwd=1.5, xaxt="n", yaxt="n", ylim=c(2, -10))
 points(bincor_Grad_MF$Binned_time_series[,1],
  bincor_Grad_MF$Binned_time_series[,2], t="l", col="gold", lwd=2.5)
 legend(bty="n", "topleft", lty=rep(1,2), lwd=c(1.5,3), col=rep(3,2),
  legend=c("Pollen time series", "Binned pollen time series"))
```

```
 legend(bty="n", "topright", lty=rep(1,2), lwd=c(1.5,3), col=rep("gold",2),
  legend=c("SST gradient time series", "Binned SST gradient time series"))
 axis(4, at=pretty(c(Grad[,2])), las=1, col="gold", col.lab="gold",
  col.axis="gold")
 mtext(4, text="SST gradient (°C)", line=2.5, col.lab=2, col="gold")
dev.off()

 ######################################################################
 bincor_d18O_MF <- bin_cor(d18O, MF, FLAGTAU=3, rho1=0.9627, tau1=12.9420,
  rho2=0.8737, tau2=3.03774, ofilename="binnedts_d18O_MF.dat")
 # Overplot
pdf("plot_MF_d18O_original_binned_ts.pdf", width=12, height=9)
 par(oma=c(0, 0, 0, 1), mar=c(4.5, 4.5, 2, 3.5) + 0.1)
 plot(MF[,1], MF[,2], t="l", xlab="Age (Ka)", ylab="MF pollen (%)",
  las=1, lwd=1.5, col=3)
 points(bincor_Grad_MF$Binned_time_series[,1],
  bincor_Grad_MF$Binned_time_series[,3], t="l", lwd=3, col=3)
 par(new=T)
 plot(c(d18O[,1]), c(d18O[,2]), xlab="Age (Ka)", ylab="", las=1, t="l",
  col=4, lwd=1.5, xaxt="n", yaxt="n", ylim=c(5, 1))
 points(bincor_d18O_MF$Binned_time_series[,1],
  bincor_d18O_MF$Binned_time_series[,2], t="l", col=4, lwd=3)
 legend(bty="n", "topleft", lty=rep(1,2), lwd=c(1.5,3), col=rep(3,2),
  legend=c("Pollen time series", "Binned pollen time series"))
 legend(bty="n", "topright", lty=rep(1,2), lwd=c(1.5,3), col=rep(4,2),
  legend=c("d18O time series", "Binned d18O time series"))
 axis(4, at=pretty(c(d18O[,2])), las=1, col=4, col.lab=4, col.axis=4)
 mtext(4, text=expression(delta^{18}*O), line=2.5, col.lab=4, col=4)
dev.off()

 ######################################################################
 bincor_Inso_MF <- bin_cor(Inso, MF, FLAGTAU=3, rho1=0.9965, tau1=56.8592,
 #bincor_Inso_MF <- bin_cor(Inso, MF, FLAGTAU=3, rho1=0.9965, tau1=12.94,
  rho2=0.8737, tau2=3.03774, ofilename="binnedts_Inso_MF.dat")
 # Overplot
pdf("plot_MF_Inso_original_binned_ts.pdf", width=12, height=9)
 par(oma=c(0, 0, 0, 1), mar=c(4.5, 4.5, 2, 3.5) + 0.1)
 plot(MF[,1], MF[,2], t="l", xlab="Age (Ka)", ylab="MF pollen (%)",
  las=1, lwd=1.5, col=3)
 points(bincor_Inso_MF$Binned_time_series[,1],
  bincor_Inso_MF$Binned_time_series[,3], t="l", lwd=3, col=3)
 par(new=T)
 plot(c(Inso[,1]), c(Inso[,2]), xlab="Age (Ka)", ylab="", las=1, t="l",
  col=1, lwd=1.5, xaxt="n", yaxt="n", ylim=c(295, 345))
 points(bincor_Inso_MF$Binned_time_series[,1],
  bincor_Inso_MF$Binned_time_series[,2], t="l", col=1, lwd=2.5)
 legend(bty="n", "topleft", lty=rep(1,2), lwd=c(1.5,3), col=rep(3,2),
  legend=c("Pollen time series", "Binned pollen time series"))
 legend(bty="n", "topright", lty=rep(1,2), lwd=c(1.5,3), col=rep(1,2),
  legend=c("Insolation time series", "Binned insolation time series"))
 axis(4, at=pretty(c(Inso[,2])), las=1, col=1, col.lab=1, col.axis=1)
 mtext(4, text="Insolation", line=2.5, col.lab=1, col=1)
dev.off()
```
